# Vasopressin but Not Oxytocin Responds to Birth Stress in Infants

**DOI:** 10.3389/fnins.2021.718056

**Published:** 2021-08-27

**Authors:** Sara Fill Malfertheiner, Evelyn Bataiosu-Zimmer, Holger Michel, Sotirios Fouzas, Luca Bernasconi, Christoph Bührer, Sven Wellmann

**Affiliations:** ^1^Department of Gynecology and Obstetrics, Hospital St. Hedwig of the Order of St. John, University Medical Center Regensburg, Regensburg, Germany; ^2^Department of Neonatology, University Children’s Hospital Regensburg (KUNO), Hospital St. Hedwig of the Order of St. John, University of Regensburg, Regensburg, Germany; ^3^Paediatric Respiratory Unit and Department of Neonatology, University Hospital of Patras, Patras, Greece; ^4^Kantonsspital Aarau, Institute of Laboratory Medicine, Aarau, Switzerland; ^5^Department of Neonatology, Charité - Universitätsmedizin Berlin, Berlin, Germany

**Keywords:** antidiuretic hormone, copeptin, oxytocin, parturition, children, stress, brain

## Abstract

**Context:**

Birth triggers a large fetal neuroendocrine response, which is more pronounced in infants born vaginally than in those born by elective cesarean section (ECS). The two related peptides arginine vasopressin (AVP) and oxytocin (OT) play an essential role in peripheral and central stress adaptation and have a shared receptor mediating their function. Elevated cord blood levels of AVP and its surrogate marker copeptin, the C-terminal part of AVP prohormone, have been found after vaginal delivery (VD) as compared to ECS, while release of OT in response to birth is controversial. Moreover, AVP, copeptin and OT have not yet been measured simultaneously at birth.

**Objective:**

To test the hypothesis that AVP but not OT levels are increased in infants arterial umbilical cord blood in response to birth stress and to characterize AVP secretion in direct comparison with plasma copeptin.

**Methods:**

In a prospective single-center cross-sectional study, we recruited healthy women with a singleton pregnancy and more than 36 completed weeks of gestation delivering via VD or ECS (cesarean without prior uterine contractions or rupture of membranes). Arterial umbilical cord blood samples were collected directly after birth, centrifuged immediately and plasma samples were frozen. Concentrations of AVP and OT were determined by radioimmunoassay and that of copeptin by ultrasensitive immunofluorescence assay.

**Results:**

A total of 53 arterial umbilical cord blood samples were collected, *n* = 29 from VD and *n* = 24 from ECS. Ten venous blood samples from pregnant women without stress were collected as controls. AVP and copeptin concentrations were significantly higher in the VD group than in the ECS group (both *p* < 0.001), median (range) AVP 4.78 (2.38–8.66) vs. 2.38 (1.79–3.88) (pmol/L), copeptin 1692 (72.1–4094) vs. 5.78 (3.14–17.97), respectively, (pmol/L). In contrast, there was no difference in OT concentrations (pmol/L) between VD and ECS, 6.00 (2.71–7.69) vs. 6.14 (4.26–9.93), respectively. AVP and copeptin concentrations were closely related (Rs = 0.700, *p* < 0.001) while OT did not show any correlation to either AVP or copeptin. In linear regression models, vaginal delivery and biochemical stress indicators, base deficit and pH, were independent predictors for both AVP and copeptin. OT was not linked to base deficit or pH.

**Conclusion:**

Vaginal birth causes a profound secretion of AVP and copeptin in infants. Whereas AVP indicates acute stress events, copeptin provides information on cumulative stress events over a longer period. In contrast, fetal OT is unaffected by birth stress. Thus, AVP signaling but not OT mediates birth stress response in infants. This unique hormonal activation in early life may impact neurobehavioral development in whole life.

## Introduction

Childbirth involves a complex hormonal action including the peptides arginine vasopressin (AVP) and oxytocin (OT) to facilitate fetal adaptation from intra- to extra-uterine life, involving cardiovascular and pulmonary adaptation, thermoregulation, metabolic homeostasis and behavior (Schorscher-Petcu et al., 2010; Wellmann and Bührer, 2012; Evers and Wellmann, 2016; Kasser et al., 2019).

AVP, also known as antidiuretic hormone, is produced as a larger precursor AVP prohormone in the hypothalamus, cleaved in different peptides, including the bioactive non-apeptide AVP and copeptin, the C-terminal part of AVP prohormone. These are eventually secreted in equimolar amounts by the posterior pituitary gland (Christ-Crain, 2019). The physiological functions of AVP, i.e., homeostasis of fluid balance, vascular tone and regulation of the endocrine stress response, are mediated by three AVP receptors, V1aR, V1bR, and V2R that are encoded for by three separate genes (Carter et al., 2020).

Similar to AVP, OT is also synthesized in the hypothalamus and differs in only two amino acids from its evolutionary older sister peptide AVP and exerts its action via the OT receptor (OTR), including patterns of growth, resilience, and healing with stress-coping, anti-inflammatory, and antioxidant functions (Robert and Clauser, 2005). However, AVP and OT bind with similar affinity to their cognate receptors OTR and V1aR.

At the end of pregnancy, the maternal level of oxytocin rises, and a noticeable surge of maternal OT occurs at parturition in the mothers blood (Ross and Young, 2009; Uvnäs-Moberg et al., 2019). However, data on human fetal OT levels are conflicting with studies reporting an increase in response to birth stress (Marchini et al., 1988; Lindow et al., 1998) while others did not (Patient et al., 1999). An array of conditions that stimulate release of AVP, such as low blood pressure, high osmolarity, hypoxia, pain, and stress, also regulate oxytocin secretion.

On top of their broad peripheral actions, both peptides AVP and OT are well known for their distinct neurobehavioral actions (Neumann and Landgraf, 2012; Carter et al., 2020). Interestingly, due to a large structural homology between the OT receptor and the main AVP receptor (V1aR), there is significant cross-reactivity between the ligands and receptors resulting in overlaps of AVP and OT peripheral and central actions (Pierce et al., 2020).

In contrast to OT, which ideally is determined in blood by OT itself, AVP concentrations in blood can be deduced from analyzing copeptin concentrations. In fact, copeptin measurement achieved wide clinical utility as copeptin is much more stable than AVP and can be determined quantitatively in an automated fashion (Morgenthaler et al., 2006; Fenske et al., 2018). Despite its use as a diagnostic marker in a large variety of diseases, the physiological function of copeptin is widely unknown (Christ-Crain, 2019).

AVP, its surrogate marker copeptin and OT were measured each in many studies on fetal response to childbirth (Wellmann et al., 2010; Evers and Wellmann, 2016). There is large agreement that birth stress causes fetal AVP and copeptin secretion in humans and other species, e.g., rodents (Evers and Wellmann, 2016; Summanen et al., 2018). However, there is no study yet published showing AVP and copeptin measurements in parallel in infants which is unlike adults (Christ-Crain, 2019).

The present study was designed to directly compare for the first time the three peptides, AVP, copeptin, and oxytocin, in arterial umbilical cord blood samples in response to birth stress. The two aims of our study were first, to assess whether fetal OT level respond to birth stress by comparing fetal arterial umbilical cord blood OT levels after vaginal delivery (VD) and elective cesarean section (ECS) and, second, to assess AVP and copeptin in parallel.

## Materials and Methods

This prospective, cross-sectional study was conducted in a University women’s and children’s hospital in Germany. The study was approved by the Ethics Committee of the University of Regensburg (file number: 20-1876-101) and written consent was obtained prior to enrollment. Inclusion criteria for both birth modes, VD and ECS, were singleton pregnancy and a gestational age of 37-42 weeks. ECS was defined as cesarean section done either on maternal request or due to medical indications, such as breech presentation or repeated cesarean. Hence, no onset of labor, meaning no uterine contractions or rupture of membranes before ECS, was mandatory. In contrast, instrumental VD and secondary cesarean (defined as cesarean section after the onset of uterine contractions, e.g., due to pathological fetal heart rate tracing) was an exclusion criterion. Further maternal exclusion criteria were infections, hypertension, preeclampsia, and substance abuse. Fetal exclusion criteria were fetal malformation, chromosomal aberration, fetal growth restriction and infections. Infants requiring advanced medical support and admitted to the neonatal intensive care unit were also excluded from the study.

In addition, venous blood samples from 10 healthy pregnant women (37-40 weeks of gestation) at regular pregnancy control visits and before the onset of uterine contractions or rupture of membranes, were used as controls. The inclusion and exclusion criteria for this group were the same as above.

Arterial umbilical blood samples (0.5 mL) were collected directly after birth into EDTA tubes and plasma was frozen at −20°C (maximum within 30 min after delivery). All samples were centrifuged by 4°C up to 10 min with a relative centrifugal force of 1,300 g according to a previously published protocol (Kagerbauer et al., 2013). AVP and OT were measured by using highly sensitive and specific radioimmunoassays (RIAgnosis, Regensburg, Germany). In detail, plasma samples were stored at −20°C until extraction using LiChroprep Si60 heat-activated at 700°C for 3 h. 20 mg of LiChroprep Si60 in 1 mL of distilled water was added to each sample, which was then mixed for 30 min, washed twice with distilled water and 0.01 N HCI and eluded with 60% acetone. The extraction recovery range was 85–90%. The data was not corrected for recovery. After evaporation, 50 μL of an assay buffer was added to the extract to assay OT and AVP using specific and sensitive radioimmunoassays. Briefly, 50 μL of an antibody was added to each sample. Following a 1-h preincubation period, 10 μL of 125I-labeled tracer was added to the sample, which was then incubated for 3 days at 4°C. Unbound radioactivity was precipitated by activated charcoal. In these environments, on average 50% of total counts are bound with < 5% non-specific binding. The detection limit is in the 0.1–0.5 pg/sample range, depending on the age of the tracer, with typical displacements of 20–25% at 2 pg, 60–70% at 8 pg, and 90% at 32 pg of standard neuropeptide.

Measurements of copeptin were done by using an ultrasensitive immunofluorescence assay (Evers and Wellmann, 2016). The laboratory staff was blinded to the aims of the study.

Information concerning maternal body mass index prior to pregnancy, gestational age at delivery, delivery parameters (umbilical blood pH and base excess-BE) and newborn characteristics at birth (weight, head circumference, length, Apgar levels) were collected.

Continuous variables (median with IQR) were compared with the Mann-Whitney *U*-test and categorical variables (number of cases, %) with the chi-square test. Spearman’s rank-order correlation was applied to assess the correlation among AVP, copeptin, and OT. Univariable linear regression analysis was used to explore the effect of delivery mode, sex, gestational age, birth weight, umbilical blood pH and base deficit (independent variables) on the log-transformed AVP, copeptin, and OT levels (dependent variables). Parameters with *P* < 0.1 in the univariable analysis were included in multivariable models. Statistical analyses were performed using the IBM SPSS version 25 (IBM Corp., Armonk, NY).

## Results

In total, 29 subjects were included in the VD group and 24 in the ECS group. There were no differences in mothers somatometric data and infants weight, head circumference, sex and postnatal adaptation characteristics between the two groups (Table 1). In contrast, delivery was on average 10 days earlier, infants length slightly smaller, and the arterial umbilical cord blood less acidotic (higher pH and/or less BE) in the ECS group (Table 1).

**TABLE 1:** Characteristics of mothers and newborns, and pregnancy outcome according to mode of delivery.

	**Vaginal delivery (*n* = 29)**	**Elective cesarean section (*n* = 24)**	***P***
Age of gestation (days)	281 (273–285)	271 (267–274)	<0.001
Mother body mass index	22.7 (20.8–24.7)	22.7 (21.1–23.9)	n.s.
Infant weight (g)	3,535 (3,238–3,668)	3,310 (2,970–3,548)	n.s.
Infant length (cm)	53 (51–55)	51 (50–53)	0.029
Infant head circumference (cm)	35 (34–37)	35 (34–36)	n.s.
Infant sex, male	17 (59)	14 (58)	n.s.
pH arterial umbilical cord blood	7.21 (7.19–7.32)	7.33 (7.31–7.36)	<0.001
BE arterial umbilical cord blood	−5.7 (−7.35 to −4.30)	−1.60 (−2.20 to −1.00)	<0.001
Apgar 5 min > 5	24 (79)	21 (88)	n.s.
Apgar 10 min > 5	29 (100)	23 (96)	n.s.

*Data are median (IQR) or number of cases (%). Body mass index prior to pregnancy. Comparisons were performed with Mann-Whitney U or chi-square test, as appropriate.*

*n.s., not significant; BE, base excess.*

**FIGURE 1:**
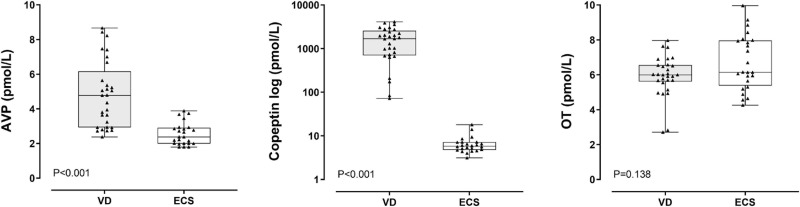
Arginine Vasopressin (AVP), copeptin and oxytocin (OT) levels according to mode of delivery. Comparisons were performed with Mann-Whitney *U*-test. VD, vaginal delivery; ECS, elective cesarean section.

AVP and copeptin levels were higher in the VD group (AVP median 4.78 pmol/L, IQR 2.93–6.16; copeptin median 1,691 pmol/L, IQR 704–2,568 pmol/L) as compared to the ECS group (AVP median 2.38 pmol/L, IQR 1.9–2.91 pmol/L; copeptin median 5.78 pmol/L, IQR 4.75–7.16 pmol/L; *p* < 0.001 for both comparisons). In contrast, OT levels were similar between the two (VD: 6.0 pmol/L, IQR 5.61–6.56 pmol/L; ECS: 6.14 pmol/L, 5.38–7.96 pmol/L) (Figure 1).

There was a strong correlation between AVP and copeptin (Spearman’s rho 0.700, *p* < 0.001) but not between AVP and OT (–0.031, *p* = 0.825) or between copeptin and OT (–0.185, *p* = 0.185). AVP and copeptin were negatively correlated with the pH (–0.603 and –0.738, respectively) and the BE (–0.597 and –0.749, respectively) (*p* < 0.001 for all correlations). There was no correlation between OT and pH or between OT and BE (data not shown).

Linear regression analyses showed that VD and umbilical cord blood acidity were both independent predictors of AVP and copeptin levels, while the OT levels were unrelated to any of the clinical or biochemical parameters (Table 2).

**TABLE 2:** Effect of various parameters on arterial umbilical cord blood levels of arginine vasopressin, copeptin, and oxytocin.

	**Unadjusted effect**		**Adjusted effect**	
	**Beta coefficient *b***	***P***	**Beta coefficient**	***p***
**AVP**				
Vaginal delivery	0.667	<0.001	0.336	0.030
Male sex	0.151	0.280	−	−
GA	0.468	<0.001	0.134	0.308
BW	0.307	0.025	0.034	0.771
Umbilical blood pH	−0.620	<0.001	−*	−
Umbilical blood BE	−0.644	<0.001	− 0.372	0.010
**Copeptin**				
Vaginal delivery	0.953	<0.001	0.758	<0.001
Male sex	0.086	0.542	−	−
GA	0.544	< 0.001	0.049	0.264
BW	0.237	0.088	–0.048	0.228
Umbilical blood pH	−0.720	<0.001	−*	
Umbilical blood BE	−0.763	<0.001	–0.276	<0.001
**OT**				
Vaginal delivery	−0.242	0.081	−0.162	0.327
Male sex	−0.035	0.803	−	−
GA	−0.235	0.090	−0.145	0.378
BW	−0.064	0.651	−	−
Umbilical blood pH	0.197	0.167	−	−
Umbilical blood BE	0.122	0.400	−	−

Linear regression analyses, using log-transformed AVP, copeptin or OT values as dependent variable. The unadjusted effect refers to the influence of each parameter separately (univariable analyses). The adjusted effect refers to the influence of each parameter after correcting for the effect of the others (multivariable analysis). Only parameters with P < 0.1 in the univariable models were included in the multivariable analysis.

*Not included in the final model due to significant collinearity with pH. Coefficients of determination (R^2^) for the multivariable models: AVP 0.529, copeptin 0.946, OT 0.073.

*AVP, arginine vasopressin; OT, oxytocin; GA, gestational age; BW, birth weight, BE, base excess.*

When comparing AVP, copeptin and OT concentrations in all infants (*n* = 53) with those of the pregnant women before the onset of labor (control group, *n* = 10), all three peptides were significantly higher in infants. More specifically, AVP was 2.93 pmol/L (median; IQR 2.38–4.92 pmol/L) in infants vs. 2.001 pmol/L (1.74–2.77 pmol/L) in controls (*P* = 0.042), copeptin 174.4 pmol/L (5.99–1831.5 pmol/L) vs. 7.44 pmol/L (5.52–9.68 pmol/L) (*p* < 0.001), and OT 6.06 pmol/L (5.59–7.16 pmol/L) vs. 4.00 pmol/L (3.69–4.26 pmol/L) (*p* < 0.001), respectively.

## Discussion

The peptides AVP and OT are key hormones in the peripheral system and in the brain with largely opposite roles in modulating stress, anxiety, and social behaviors (Neumann and Landgraf, 2012). However, due to high sequence homology between both peptides and their receptors, there are many signaling interactions (Jurek and Neumann, 2018) but the conditions of their crosstalk remain in general unclear (Rae et al., 2021) and particularly in the process of childbirth (Wellmann and Bührer, 2012).

To clarify the effect of birth stress on the secretion of these peptides, the present study aimed to assess for the first time simultaneously the levels of AVP, OT, and the AVP surrogate marker copeptin in fetal arterial umbilical cord blood at birth. The main finding was that in contrast to OT, both AVP and copeptin are significantly increased after VD as compared to deliveries without preceding birth stress, namely ECS. Thus, OT secretion in infants is not subject to birth stress.

Determination of AVP and OT levels in clinical settings is challenging, as half time of both peptides is short and in particular their stability in sampling matrices is very low (Morgenthaler et al., 2006; Muttenthaler et al., 2010). In contrast to AVP, for which a more stable surrogate marker has been identified and developed for clinical routine, namely copeptin, there is no such alternative for OT. Thus, we set up a strict sampling protocol to achieve fast and reliable processing in all analytes for this study. The results indicate twofold higher AVP values in VD as compared to ECS and almost 300-fold higher copeptin values in VD as compared to ECS. These magnitudes for AVP and copeptin are in line with previous reports as summarized by Evers and Wellmann (2016). However, we are the first presenting AVP and copeptin data from same samples at birth. There was an average twofold increase of AVP associated with birth while copeptin increased more than 200-fold. As AVP has a very short half-life compared with copeptin and both are released at equimolar amounts, we assume that AVP is repetitively released with each uterine contraction but degraded in the blood soon thereafter, while copeptin accumulates.

Since we strictly followed previously published protocols on sampling and post-sampling handling with immediate cooling down to preserve stability of all peptides (Kagerbauer et al., 2013), we conclude that the observed huge difference noted between AVP and copeptin concentrations in umbilical cord blood when comparing VD and ECS is most likely caused due to a difference in half-life of AVP and copeptin once secreted into the circulatory system. Based on our results, the half-life differs by a factor up to 100. Assuming for AVP a half-life of few minutes in the circulatory system (Morgenthaler et al., 2006), that of copeptin is in the range of hours. This fits very well to the different stress levels fetuses experience during VD and ECS (Evers and Wellmann, 2016). Therefore we propose the following concept: The fetus secretes AVP and copeptin from the pituitary gland in a 1:1 ratio into the circulatory system upon relevant uterine contractions. Whereas AVP is cleared within minutes, copeptin accumulates over time. Thus, AVP level reflects short-range stress and that of copeptin represents the sum of all stress events summoned over a long period of many hours.

In the present analysis, oxytocin levels were completely unaffected by the mode of delivery and did not show any association to clinical and biochemical stress indicators nor to AVP or copeptin. This confirms previous findings by Patient et al. (1999) and supports the notion that AVP but not OT is subject to fetal birth stress (Wellmann and Bührer, 2012). Thus, in respect to fetal stress we conclude that AVP is the driver in mediating peripheral and central response supporting infants transition from intra-uterine to extra-uterine life (Evers and Wellmann, 2016; Spoljaric et al., 2017). Regarding the maternal part, OT is a key hormone in childbirth, and synthetic oxytocin is widely administered to induce labor (Uvnäs-Moberg et al., 2019). In fact, mild uterine contractions are sufficient to trigger fetal copeptin release (Wellmann et al., 2016) and a large multicenter randomized controlled trial is underway to study the clinical effect of mild uterine contractions prior to ECS on infants and mothers outcome after birth (Wellmann et al., 2021).

Animal models support the findings of a perinatal surge in AVP and copeptin with higher levels in plasma after a vaginal than a Cesarean section (Hoffiz et al., 2021). In parallel to the increase in the peripheral blood circulation also central *AVP* mRNA levels and AVP protein levels rise significantly at birth (Spoljaric et al., 2017; Hoffiz et al., 2021). Hypoxic events, which in human fetuses accompany regularly uterine contractions before birth, have been identified in animal models as large driver of AVP and copeptin release into the peripheral blood circulation and in specific brain regions (L’Abate et al., 2013; Coldren et al., 2017; Summanen et al., 2018). Whereas increased AVP levels in plasma were found to be associated with decreased plasma osmolality after a vaginal, but not Cesarean section birth (Hoffiz et al., 2021), central AVP increase is related to reduced neuronal cell death in specific brain areas (Hoffiz et al., 2021) and suppress energetically expensive correlated network events under conditions of reduced oxygen supply at birth (Spoljaric et al., 2017). To what extent peripheral or central AVP mechanisms may contribute to birth related natural analgesia in human neonates is under discussion (Kasser et al., 2019).

Strengths of our study include the prospective design, the strict adherence to sampling and post-sampling protocols as well as state of the art measurement of peptides. Limitations include the lack of maternal blood samples in parallel to that from fetuses and the lack of additional measurements within the first hours of life to determine the half-life of AVP and copeptin in the peripheral circulation of neonates.

In conclusion, AVP and copeptin reflect both birth stress but whereas AVP indicates acute stress events, copeptin provides information on cumulative stress events over a longer period. In contrast to AVP and copeptin, OT level in infants at birth are not associated with any clinical or biochemical stress indicator, thus, OT is not subject to fetal stress response during birth.

## Data Availability Statement

The raw data supporting the conclusions of this article will be made available by the authors, without undue reservation.

## Ethics Statement

The studies involving human participants were reviewed and approved by the Ethics Committee of the University of Regensburg. Written informed consent to participate in this study was provided by the participants’ legal guardian/next of kin.

## Author Contributions

SFM, EB-Z, LB, and HM performed clinical data acquisition, biomarker analyses, and coordination and check. SF performed statistical analysis and contributed to the manuscript. SFM, CB, and SW supervised the whole project. All authors made substantial contributions to conception and design, analyses and interpretation of data, and revising the article, read the manuscript, and approved its submission.

## Conflict of Interest

The authors declare that the research was conducted in the absence of any commercial or financial relationships that could be construed as a potential conflict of interest.

## Publisher’s Note

All claims expressed in this article are solely those of the authors and do not necessarily represent those of their affiliated organizations, or those of the publisher, the editors and the reviewers. Any product that may be evaluated in this article, or claim that may be made by its manufacturer, is not guaranteed or endorsed by the publisher.
